# The pro-apoptotic actions of 2-methoxyestradiol against ovarian cancer involve catalytic activation of PKCδ signaling

**DOI:** 10.18632/oncotarget.27760

**Published:** 2020-10-06

**Authors:** Purab Pal, Karen Hales, Dale Buchanan Hales

**Affiliations:** ^1^Department of Physiology, Southern Illinois University, Carbondale, IL 62901, USA; ^2^Department of Obstetrics and Gynecology, Southern Illinois University School of Medicine, Springfield, IL 62702, USA

**Keywords:** 2-methoxyestradiol, ovarian cancer, protein kinase Cδ, p38 MAPK, apoptosis

## Abstract

Background: 2-methoxyestradiol (2MeOE_2_) is a natural metabolite of estradiol, which is generated by the action of CYP1A1 enzyme in the liver. We have previously shown that a flaxseed-supplemented diet decreases both the incidence and severity of ovarian cancer in laying hens, also induces CYP1A1 expression in liver. Recently, we have shown that as a biologically derived active component of flax diet, 2MeOE_2_ induces apoptosis in ovarian cancer cells which is partially dependent on p38 MAPK. The objective of this study was to elucidate the molecular mechanism of actions of 2MeOE_2_, a known microtubule disrupting agent, in inducing apoptosis in ovarian tumors.

Results: 2MeOE_2_ induces γH2Ax expression and apoptotic histone modifications in ovarian cancer cells, which are predicted downstream targets of protein kinase Cδ (PKCδ) during apoptosis. Overexpressing full length PKCδ alone does not induce apoptosis but potentiates 2MeOE_2_-mediated apoptosis. C3-domain mutated dominant-negative PKCδ (PKCδ^DN^) significantly reduces 2MeOE_2_-induced caspase-3 cleavage and apoptotic histone modification. Silencing PKCδ diminishes 2MeOE_2_-mediated apoptosis. The catalytic fragment of PKCδ (PKCδ^CAT^) evokes pro-apoptotic effects which are principally dependent on p38 MAPK phosphorylation.

Conclusions: The pro-apoptotic actions of 2MeOE_2_ are in part dependent on catalytic activation of PKCδ. Catalytic activation of PKCδ accelerates the 2MeOE_2_-induced apoptotic cascade. This study describes a novel molecular action of flaxseed diet in ovarian cancer.

## INTRODUCTION

With an estimated 22,530 cases reported and 13,980 estimated deaths in the year 2019, ovarian cancer is the deadliest gynecological disease accounting for more deaths than any other cancer in the female reproductive tract. The disease is often diagnosed at an advanced stage, which contributes to a low five-year survival rate of only 47.6% in the United States [1].

Our laboratory studies epithelial ovarian cancer (EOC) in laying hens, the only known natural animal model that spontaneously develops the disease over its lifespan. The disease in hens is very similar to the human form in expression of similar molecular markers such as CA-125 and e-cadherin, and symptoms such as accumulation of ascitic fluid and peritoneal metastasis in the advanced stage. Onset of EOC in laying hens is also positively correlated with the number of lifetime ovulation. Suppressing ovulation reduces ovarian cancer incidence in laying hens, similar to the preventative effects of reduced ovulation observed in women.

Our research has shown that dietary ingestion of flaxseed reduces the onset and severity of ovarian cancer in laying hens [2, 3]. Flaxseed is one of the richest plant sources of omega-3 polyunsaturated fatty acids (OM3FA), mostly α-linoleic acid (ALA), phytoestrogen lignans, namely secoisolariciresinol diglucoside (SDG) and both soluble and insoluble fibers. ALA is converted to docosahexaenoic acid (DHA) by action of desaturase and elongase enzymes. DHA has potent anti-inflammatory actions by regulating Nuclear Factor kappa B (NFκB) activation and cyclooxygenase 2 (COX-2) expression (manuscript in preparation).

Estradiol is metabolized by three cytochrome P450 (CYP) enzymes in the liver. We have also shown that the flaxseed diet in hens induces CYP1A1 expression in the liver while suppressing both CYP1B1 and CYP3A4 expressions. The upregulation of CYP1A1 parallels the increase in 2hydroxyestradiol and the 2MeOE_2_ level in the serum of the chickens [4]. 2MeOE_2_ has established anti-proliferative and pro-apoptotic properties [5, 6] that have been tested on various cancer cells [7–13], although its molecular mechanisms are yet to be fully understood.

Recently we have shown that 2MeOE_2_ induces apoptosis in human ovarian cancer cells. The pro-apoptotic and anti-angiogenic effects of 2MeOE_2_ are dependent on the p38 MAPK pathway [14]. In a time-course study, caspase-3 cleavage was first detected at 24 h and TUNEL positive staining followed [14, 15]. This prompted us to probe the 2MeOE_2_ -induced apoptosis, especially the apoptotic histone modifications. Among the multiple upstream activators which triggers these histone marks during apoptosis, protein kinase Cδ (PKCδ) is one common factor. Therefore, we investigated whether the pro-apoptotic actions of 2MeOE_2_ involve PKCδ signaling.

The objective of this study was to investigate the epigenetic modifications exerted by 2MeOE_2_ and assess the role of PKCδ in 2MeOE_2_ actions in order to gain an in-depth mechanistic understanding of its molecular and cellular pro-apoptotic actions.

## RESULTS

### 2MeOE_2_ increases γH2Ax and phH3ser10 expression in human ovarian cancer cells

**Figure 1:**
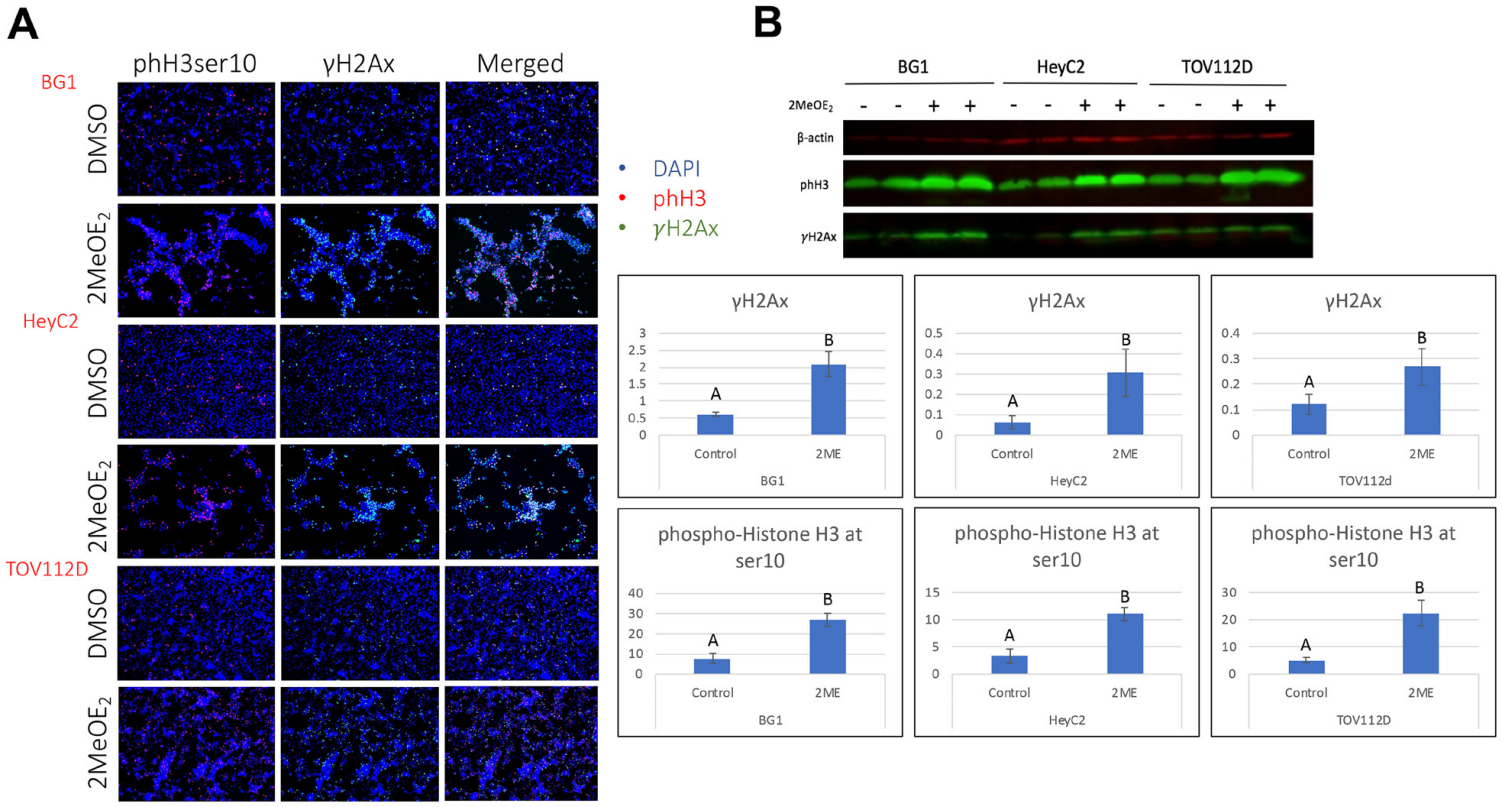
2MeOE_2_ induced histone modifications in human ovarian cancer cells. BG1, HeyC2 and TOV112D cells were treated with 10 μM 2MeOE_2_ for 24 h. (**A**) Control and 2MeOE_2_-treated cells seeded on coverslip were then immunostained with anti-γH2Ax co-stained with anti-phospho histone H3 (ser10). (**B**) Western blot for γH2Ax and phospho histone H3 (ser10) from whole cell lysates (*n* = 4). Student’s unpaired *t*-test, error bars: SEM, *p* < 0.05.

We hypothesized that 2MeOE_2_ treatment results in apoptotic histone modifications. Therefore, we investigated phospho histone H2A at ser139 (γH2Ax) and phospho histone H3 at ser10 (phH3ser10) expressions in human ovarian cancer cells after 10 μM 2MeOE_2_ treatment for 24 hours. 2MeOE_2_ treatment increased the number of cells expressing γH2Ax, a marker for DNA damage, and phH3ser10 in all three cell lines. Co-immunostained sections suggested that γH2Ax and phH3ser10 are co-expressed by a number of cells following 2MeOE_2_ treatment (Figure 1A). In order to verify our observation and to confirm that this upregulation is not because of a reduction in total number of viable cells, we quantified γH2Ax and phH3ser10 expression by western blot on total protein isolated from cells following 2MeOE_2_ treatment. The expression of γH2Ax and phH3ser10 significantly increases following 2MeOE_2_ treatment (Figure 1B).

### 2MeOE_2_ increases phH2Bser14 expression in human ovarian cancer cells

**Figure 2:**
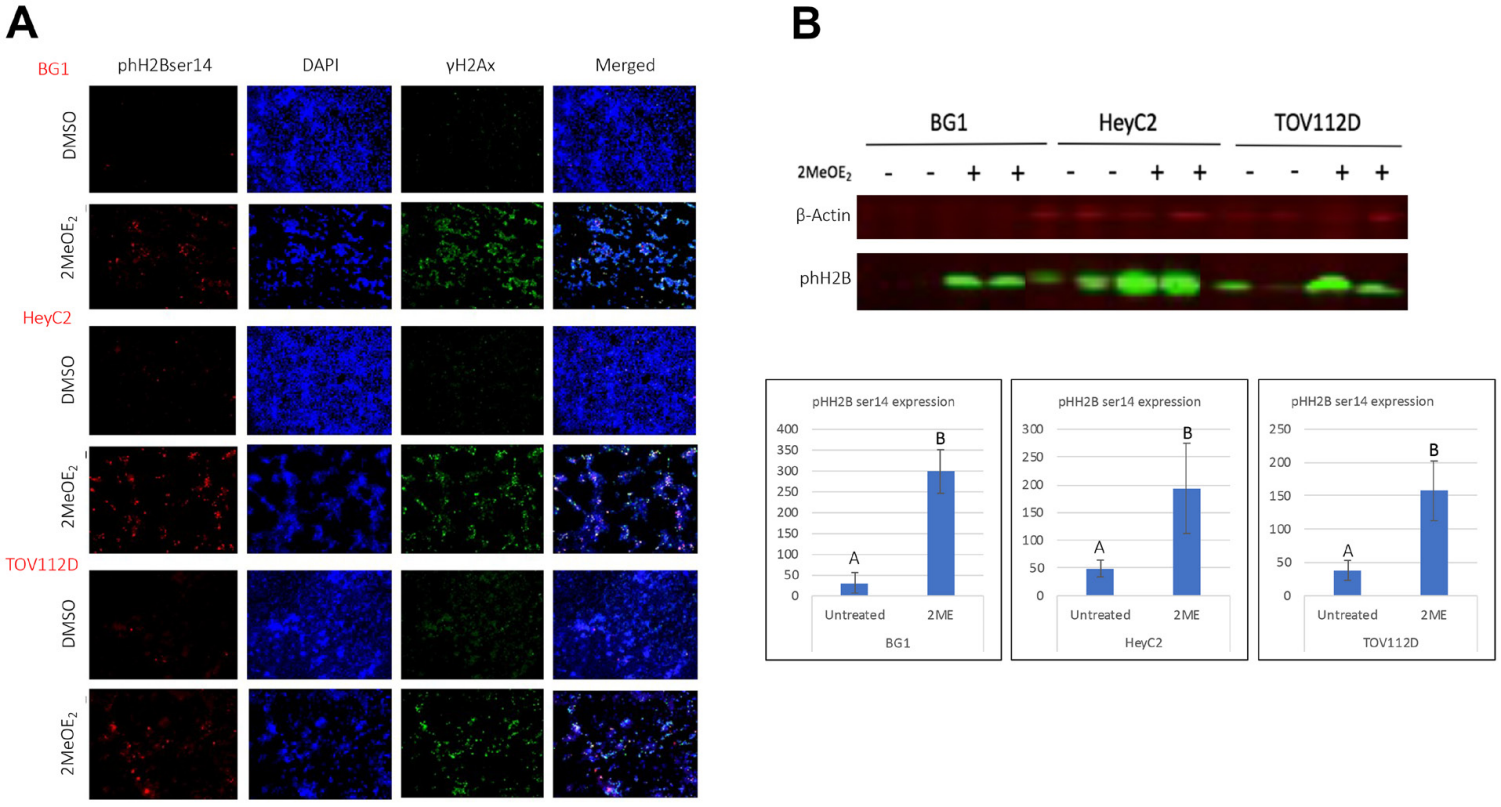
2MeOE_2_ induces phosphorylation on ser 14 of histone H2B in human ovarian cancer cells. BG1, HeyC2 and TOV112D cells were seeded on coverslips and treated with 10 μM 2MeOE_2_ for 24 h. (**A**) Control and 2MeOE_2_-treated cells were immunostained with anti-phospho histone H2B (ser14) co-stained with anti-γH2Ax. (**B**) Western blot from whole cell lysates for phospho histone H2B (ser14) (*n* = 4). Student’s unpaired *t*-test, error bars: SEM, *p* < 0.05.

To examine whether 2MeOE_2_ treatment induces apoptotic histone modification, we investigated phospho histone H2B at ser14 (phH2Bser14) expression after 2MeOE_2_ treatment. Cells were seeded on coverslips and harvested after a 24 h treatment with 10 μM 2MeOE_2_. 2MeOE_2_ treatment increased number of phH2Bser14 expressing cells in all three cell lines. Co-immunostaining against phH2Bser14 and γH2Ax revealed that similar to our earlier observation with phH3ser10, a number of cells co-expressed phH2Bser14 and γH2Ax, the marker for DNA damage (Figure 2A). Western blot data on total protein lysates confirmed significant upregulation of phH2Bser14 following 2MeOE_2_ treatment (Figure 2B).

### PKCδ^WT^ increases pro-apoptotic actions of 2MeOE_2_

**Figure 3:**
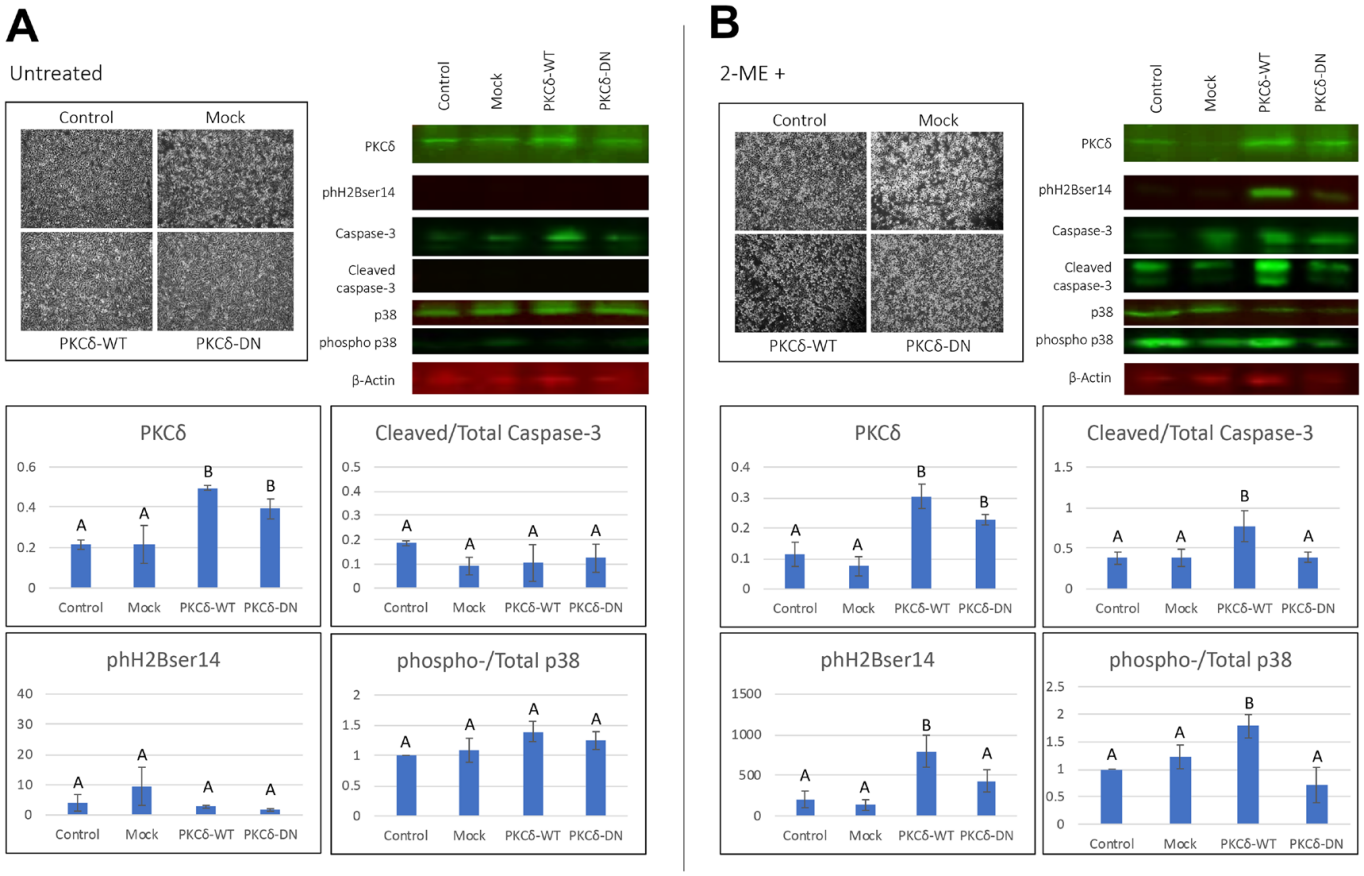
Effect of transient transfection of PKCδ^WT^ and PKCδ^DN^ to human ovarian cancer cells. (**A**) BG1 cells were either untransfected (control) or transfected with a pGFP expression vector (mock), a wild type PKCδ expression vector (PKCδ^WT^) or a C3-domain mutated PKCδ expression vector (PKCδ^DN^). (**B**) 10 μM 2MeOE_2_ was treated to BG1 cells that were either untransfected (control) or transfected with a GFP (mock control), PKCδ^WT^ or PKCδ^DN^ expression vector for 24 h. Cells were photographed and harvested after 24 h from transfection. Western blot analysis performed on total protein lysates from untransfected, mock, PKCδ^WT^ and PKCδ^DN^ transfected BG1 cells against PKCδ, phospho histone H2B (ser 14), caspase-3, cleaved caspase-3, total p38 and phospho-p38 (*n* = at least 3 for each dataset). Ratio of normalized phospho-p38 to normalized total p38 was quantified to estimate p38 phosphorylation resulting from individual transfection. Similarly, ratio of normalized cleaved caspase-3 to normalized pan caspase-3 was quantified to estimate amount of activated caspase-3. One-way ANOVA, error bars: SEM, *p* < 0.05.

Histone modifications such as phH3ser10 and phH2Bser14, have been reported to be mediated by PKCδ during apoptosis. Therefore, we hypothesized that 2MeOE_2_-mediated apoptosis in the ovarian cancer cells may involve PKCδ signaling. To address this question, BG1 cells were transfected with a wild type PKCδ (PKCδ^WT^) or a kinase-negative PKCδ (PKCδ^DN^). BG1 cells transfected with a pGFP expression vector was taken as a transfection control (mock). Cells showed no apparent changes in total numbers or gross morphology following the transfections (Figure 3A). Western blot on total cellular lysates confirmed that the amount of Pδ^WT^ and PKCδ^DN^ were increased after the transfection. PKCδ^WT^ or PKCδ^DN^ did not induce cleavage of caspase-3 or phosphorylation of p38 MAPK. Also, neither PKCδ^WT^ nor PKCδ^DN^ altered phH2Bser14 expression in the BG1 cells (Figure 3A).

Although PKCδ^WT^ or PKCδ^DN^ did not induce or prevent apoptosis alone, PKCδ^WT^ significantly potentiated the pro-apoptotic actions of 2MeOE_2_. BG1 cells transfected with PKCδ^WT^, PKCδ^DN^ or pGFP (mock) were treated with 10 μM 2MeOE_2_ for 24 h. Western blots demonstrated that 2MeOE_2_ treatment of BG1 cells transfected with PKCδ^WT^ resulted in a significantly higher amount of cleaved caspase-3, phosphorylated p38 MAPK and phH2Bser14 expression compared to untransfected and mock transfected BG1 cells. Notably, 2MeOE_2_ treatment of the PKCδ^DN^ -transfected cells did not alter cleavage of caspase-3, phosphorylation of p38 MAPK or phH2Bser14 expression compared to the untransfected and mock (Figure 3B).

### Knock down of PKCδ decreases 2MeOE_2_ mediated apoptosis in BG1 cells

**Figure 4:**
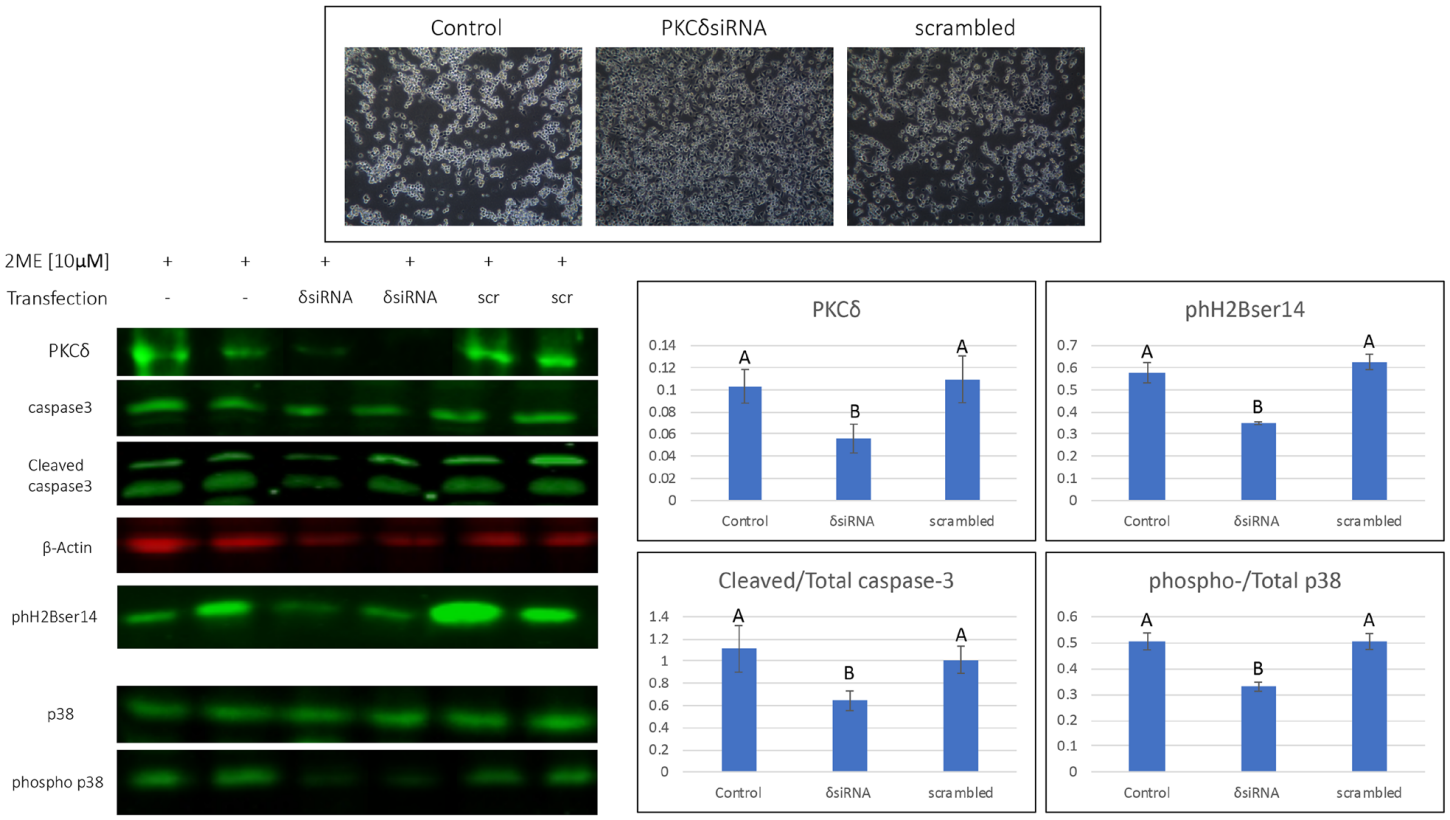
2MeOE_2_ effects on human ovarian cancer cells after silencing PKCδ. BG1 cells were either untransfected or transfected with a PKCδ siRNA or scrambled shRNA expression vectors, before treating them with 10 μM 2MeOE_2_. Cells were photographed after a 24 h incubation in 2MeOE_2_. Western blot analysis performed on total protein lysates against PKCδ, cleaved and total caspase-3, phospho histone H2B (ser 14), total and phosphorylated p38 (*n* = 4 for each dataset). One-way ANOVA, error bars: SEM, *p* < 0.05.

To investigate the role of PKCδ in 2MeOE_2_ -mediated apoptosis in the ovarian cancer cells, we performed a siRNA-mediated knockdown of PKCδ. BG1 cells were transfected with a PKCδ^RNAi^ expression vector to knock down endogenous PKCδ. BG1 cells transfected with a plasmid expressing scrambled shRNA was used as a silencing control. PKCδ^RNAi^ -transfected cells were visibly more resistant to 2MeOE_2_ treatment compared to the untransfected and scrambled shRNA transfected cells (Figure 4). Western blots confirmed the decrease of PKCδ in BG1 cells. In the PKCδ^RNAi^ transfected cells, 2MeOE_2_ treatment had significantly decreased cleavage of caspase-3 and phosphorylation of p38 MAPK and phH2Bser14 expression, compared to the untransfected and scrambled shRNA transfected cells (Figure 4).

### PKCδ^CAT^ parallels some 2MeOE_2_ induced effects, induces apoptosis in a p38-dependent manner

**Figure 5:**
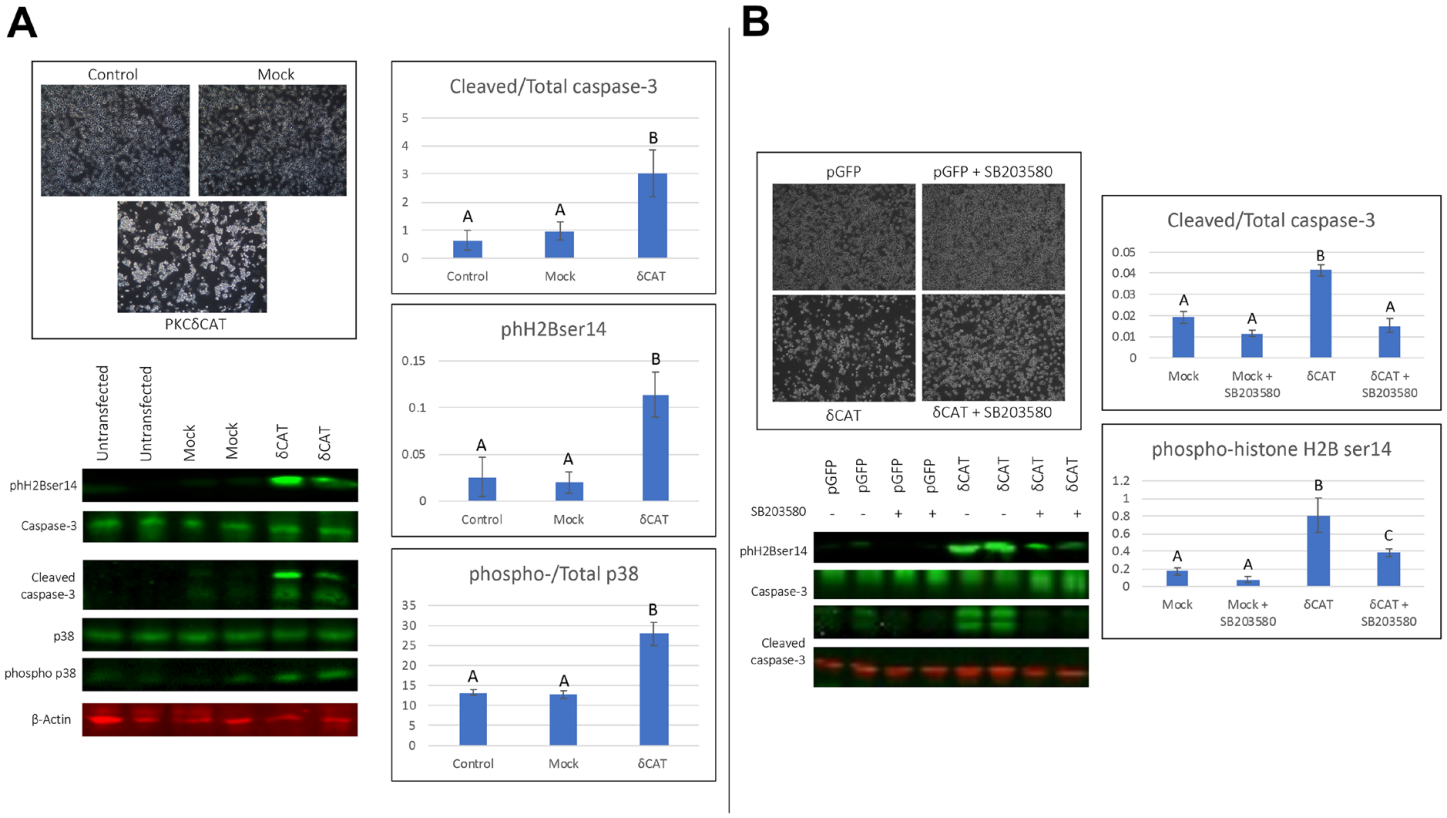
Expression of the catalytically active fragment of PKCδ to human ovarian cancer cells. (**A**) BG1 cells were either untransfected or transfected with a GFP expression vector (mock) or an expression vector with the catalytically active fragment of PKCδ (PKCδ^CAT^). Cells were photographed and harvested after 24 h of transfection. Western blot analysis on total cell lysates against cleaved and total caspase-3, phospho histone H2B (ser 14), total and phosphorylated p38 (*n* = 4 for each dataset). One-way ANOVA, error bars: SEM, *p* < 0.05. (**B**) BG1 cells were transfected with either a GFP expression vector (mock) or an expression vector with catalytically active fragment of PKCδ (PKCδ^CAT^) with or without 10 μM SB203580, a p38 MAPK inhibitor. Cells were photographed after 24 h of transfection. Western blot analysis on total cell lysates against phospho histone H2B (ser 14), cleaved and total caspase-3 (*n* = 3 for each dataset). Two-way ANOVA, error bars: SEM, *p* < 0.05.

PKCδ can be activated by either phosphorylation leading to structural changes in the full length molecule or catalytic cleavage by caspases which liberates its catalytic fragment (PKCδ^CAT^). To investigate the role of PKCδ^CAT^ in the pro-apoptotic effects, we tested whether overexpression of PKCδ^CAT^ alone can exert the 2MeOE_2_ -mediated pro-apoptotic effects. BG1 cells were transfected with the PKCδ^CAT^ expression vector and pGFP transfected cells were used as transfection controls. Following 24 h of transfection, PKCδ^CAT^ transfected cells were visibly fewer in number and had more floating cells compared to the untransfected and the mock control (Figure 5A). Western blots demonstrated the cleavage of caspase-3, phosphorylation of p38 MAPK and phH2Bser14 expression in the PKCδ^CAT^ transfected cells were significantly higher than the controls (Figure 5A). In summary, PKCδ^CAT^ significantly induced apoptosis in the BG1 cells and the effects were similar to that exerted by 2MeOE_2_.

To test whether the activation of p38 MAPK by PKCδ^CAT^ is associated with its pro-apoptotic effects, BG1 cells were transfected with a pGFP or PKCδ^CAT^ and treated with +/− 10 μM SB203580, a selective p38 MAPK inhibitor. SB203580 had no visible effect on pGFP transfected cells, but it reduced the number of rounded up and floating cells that were transfected with PKCδ^CAT^ (Figure 5B). Western blots demonstrated that SB203580 reduces phH2Bser14 expression and cleavage of caspase-3 in the PKCδ^CAT^ transfected BG1 cells, while having no significant effect in the pGFP transfected cells. Notably, SB203580 treated PKCδ^CAT^ cells had a significantly higher phH2Bser14 expression compared to its pGFP control (Figure 5B).

### PKCδ^DN^ partially reverses 2MeOE_2_ induced pro-apoptotic effects

**Figure 6:**
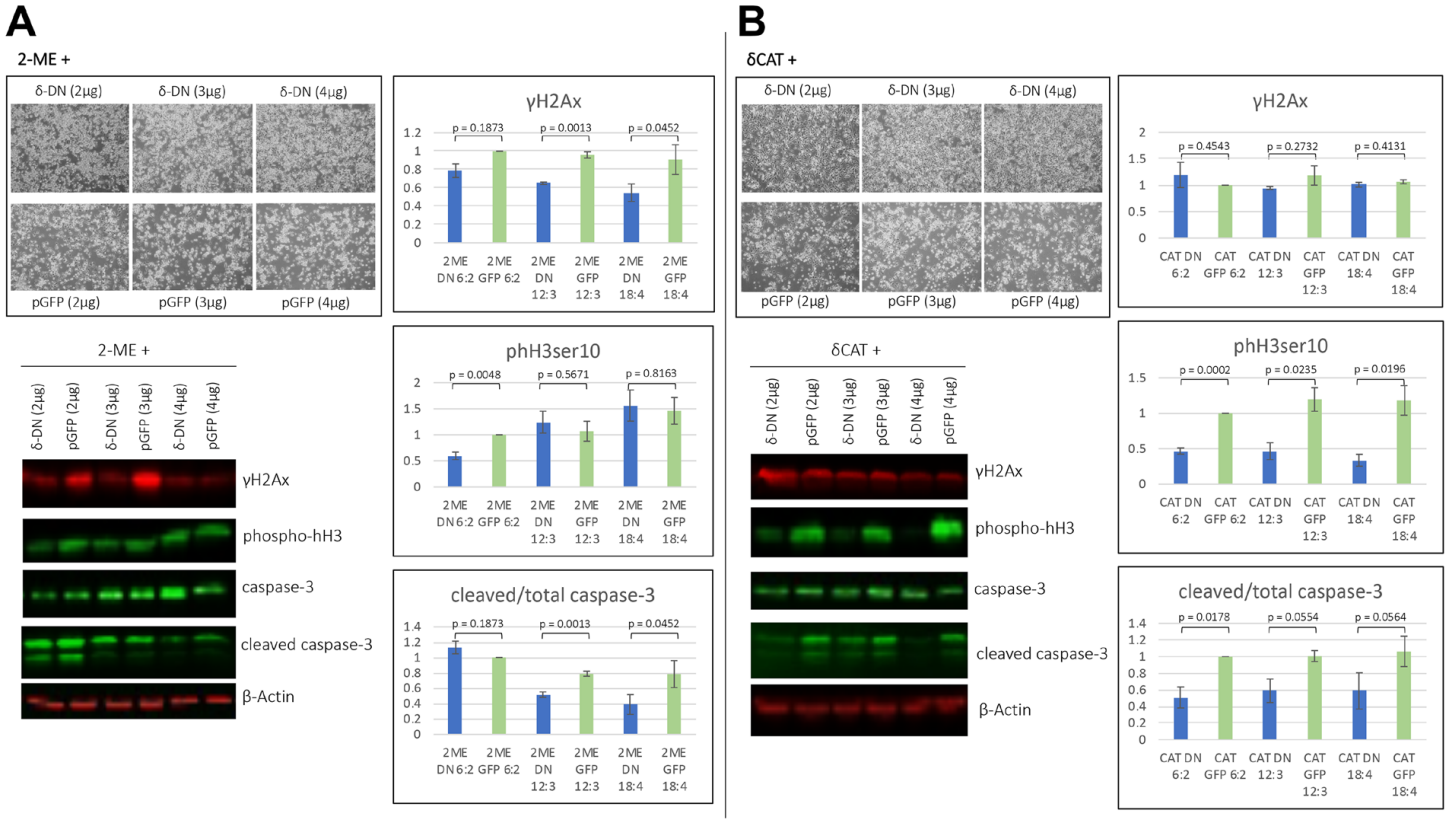
2MeOE_2_ effects are in part dependent on PKCδ. (**A**) BG1 cells were transfected with a fixed amount (2 μg) of PKCδ^CAT^ and increasing amount (2 μg, 3 μg, and 4 μg) of either PKCδ^DN^ or pGFP (mock). Cells harvested and photographed after 24 h of transfection. Western blot analysis on cell lysates against γH2Ax, phospho histone H3 (ser10), total and cleaved caspase-3 (*n* = 3 for each dataset). Student’s unpaired *t*-test, error bars: SEM, *p* < 0.05. (**B**) BG1 cells were transfected with increasing amount (2 μg, 3 μg, and 4 μg) of PKCδ^DN^ or pGFP (mock). After 24 h of transfection, cells were treated with 10 μM 2MeOE_2_ for next 24 h, photographed and harvested. Western blot on total cell lysates against γH2Ax, phospho histone H3 (ser10), total and cleaved caspase-3 (*n* = 3 for each dataset). Student’s unpaired *t*-test, error bars: SEM, *p* < 0.05.

To test whether the pro-apoptotic effects of 2MeOE_2_ are dependent on PKCδ, BG1 cells were transfected with an increasing amount of PKCδ^DN^ (2 μg, 3 μg and 4 μg of plasmid DNA) with same amount of respective pGFP controls. After 24 h of transfection, 10 μM 2MeOE_2_ was added to the cells for another 24 h. Western blot on total protein lysates indicated that higher amounts of PKCδ^DN^ (3 μg and 4 μg) significantly reduces 2MeOE_2_ -induced γH2Ax expression and cleavage of caspase-3 compared to their respective pGFP controls. phH3ser10 expression was decreased in 2 μg PKCδ^DN^ transfected cells, however there was no change observed in 3 μg and 4 μg PKC^DN^ transfected groups (Figure 6A).

To examine the PKCδ-specific effects, we transfected BG1 cells with PKCδ^CAT^ and co-transfected with an increasing amount (2 μg, 3 μg and 4 μg) of PKCδ^DN^ or pGFP as respective controls. Western blot on the total protein lysates indicated that PKCδ^DN^ has no effect on γH2Ax expression, but phH3ser10 and cleaved caspase-3 expressions were found to be significantly decreased in all PKCδ^DN^ transfected groups compared to their respective pGFP controls (Figure 6B). These data suggest PKCδ augments 2MeOE_2_ -induced DNA damage response, resulting in apoptosis. However, no consistent trend was observed in phH3ser10 expression upon PKCδ^DN^ treatment possibly because phH3ser10 is also expressed in multiple other events such as mitosis, oxidative stress, etc.

## DISCUSSION

2MeOE_2_ is a natural metabolite of estradiol (E_2_) that has known anti-angiogenic and anti-tumor activity. Anti-proliferative effects of 2MeOE_2_ have been extensively studied in ovarian cancer [16, 17], melanoma [18, 19], lung cancer [20–23], breast cancer [24–28], prostate cancer [29–31] and several other cancers [7, 9, 32, 33]. According to the current understanding, the anti-tumor actions of 2MeOE_2_ are accomplished by binding at/near the colchicine binding sites of the growing microtubules [34]. This suppresses the microtubule dynamics resulting in a G_2_/M phase arrest, and subsequent phosphorylation of Bcl_2_ [31, 35, 36] and Bcl-xL [37], preventing their anti-apoptotic activity. 2MeOE_2_ has also been reported to inhibit the expression, nuclear retention and transcriptional activity of hypoxia-inducible factor 1α (HIF-1α) and therefore preventing hypoxia-driven angiogenesis [38]. However, the cellular and molecular mechanism of action of 2MeOE_2_ is not thoroughly understood. In addition to disrupting microtubule dynamics, 2MeOE_2_ has been reported to activate a number of cellular kinases and several pro-apoptotic factors. Recently, we have reported that 2MeOE_2_ induces apoptosis in human ovarian cancer cells, which is partially dependent on activation of p38 MAPK. The current study was designed to gain a comprehensive understanding of the molecular actions of 2MeOE_2_ in ovarian cancer cells.

In the current study, we have extended our investigation to the epigenetic histone modifications induced by 2MeOE_2_. Histones were originally thought to be simple static scaffolds that package the DNA of a cell. However, it is well established now that the histones are dynamic proteins that undergo critical post-translational modifications for proper chromatin function [39]. The fate of a cell is dependent on distinct combination of histone modifications [40–42]. The *Histone Code* hypothesis proposed by Allison and Turner [43] states that not a single histone modification mark, but a collective combination of different histone modifications code for the fate of the cell. The *apoptotic histone code* includes severe dephosphorylation in the H1 linker region [44–46] and a number of phosphorylation, acetylation and methylation events on different histone monomers [47], as illustrated in Supplementary Figure 1A.

2MeOE_2_ treatment of human ovarian cancer cells causes increased phospho histone H3 at ser10 (phH3ser10) expression. Phosphorylation at this site has been traditionally regarded as a mitotic marker which correlates with chromosome condensation during mitosis and meiosis [48]. Therefore, phH3ser10 is classically associated with cell division and proliferation. On the other hand, growing evidence suggests that cells undergoing apoptosis after being exposed to various death stimuli also express phH3ser10. Therefore, this epigenetic mark is associated with chromosome compaction but the specific cellular effects of this phosphorylation is unclear. When we have co-immunostained 2MeOE_2_ treated human ovarian cancer cells with phH3ser10 and phospho histone H2A at ser139 (γH2Ax), a known DNA damage marker, we have observed that some cells expressing phH3ser10 are also undergoing DNA damage. A growing body of evidence suggests that distinctly different upstream kinases phosphorylate histone H3 at ser 10 under different conditions. During mitosis, this phosphorylation is mediated by mitotic kinases such as Aurora B and Vaccinia-related kinase 1 (VRK1) [49], whereas during apoptosis, the same phosphorylation is mediated by protein kinase Cδ (PKCδ) [50].

Similar to the phH3ser10 mark, phosphorylation of histone H2B at ser 14 (phH2Bser14) was also upregulated following 2MeOE_2_ in human ovarian cancer cells. Most of the phH2Bser14 positive cells were also positive for γH2Ax, indicative of DNA damage. The phH2Bser14 mark is associated with apoptosis whereas acetylation of the adjacent lysine residue (K15) is a property of surviving cells [51]. Acetylated K15 mark in surviving cells prevents phosphorylation in the ser 14 residue, which is deacetylated during apoptosis [52]. The ser 14 site of histone H2B is known to be phosphorylated by both Mammalian sterile 20 kinase (Mst-1) [53] and PKCδ [54, 55] during apoptosis. Interestingly, both of these enzymes are activated by caspase-3-mediated cleavage. Taken together, from the observations that 2MeOE_2_ induces both phH3ser10 and phH2Bser14 in the cells undergoing DNA damage, we hypothesized that PKCδ, being a common activator of both histone modifications, potentially plays a role in 2MeOE_2_ induced apoptosis.

The full-length human PKCδ has a catalytic domain which is essential for its enzymatic activity. The catalytic domain has a C3 (ATP binding domain) and a C4 (substrate binding) domain [56]. PKCδ also has a regulatory domain which contains two constant regions (C1 and C2-like) and a pseudo-substrate region, that keeps the full length PKCδ in a folded in an inactive conformation, preventing access to the substrate-binding pocket (C4 domain) [56, 57]. The catalytic and regulatory domains are connected by a hinge region (as described in Supplementary Figure 1B). Several apoptotic agents can induce a caspase-mediated cleavage of full-length PKCδ. This liberates the catalytic fragment (PKCδ^CAT^) which is capable of inducing chromatin condensation and DNA damage leading to apoptosis [58–60]. The PKCδ^CAT^ can freely translocate to nucleus and/or mitochondria [61–63] and promote apoptosis [60, 64, 65]. PKCδ is also suggested to interact with c-Abl tyrosine kinase. Phosphorylation at the tyrosine residues in the C4 domain mediated by c-Abl have been found to induce the nuclear translocation of PKCδ [66], although the exact mechanism of nuclear transport of PKCδ is largely unknown.

In the current study, we have seen that overexpressing the full-length PKCδ (PKCδ^WT^) and a C3 domain-mutated (K376R) PKCδ (PKCδ^DN^) transfection in the BG1 cells had no effect on apoptosis, suggesting that the full-length PKCδ needs to be activated to trigger apoptosis. After cells were treated with 2MeOE_2_, the PKCδ^WT^ transfected cells evoked a greater apoptotic response compared to the mock control and the PKCδ^DN^ transfected cells. This suggested that the 2MeOE_2_ treatment activates full-length PKCδ which triggers the apoptotic cascade. Following 2MeOE_2_ treatment, only the PKCδ^WT^ transfected cells showed significantly higher levels of phH2Bser14 expression, suggesting that the PKCδ activation can induce this epigenetic histone modification. We have also observed that a siRNA mediated PKCδ depletion made the BG1 cells significantly more resistant towards 2MeOE_2_-mediated apoptosis. PKCδ knockdown also reduced the phH2Bser14 expression, paralleling the cleavage of caspase-3.

These findings indicate that the 2MeOE_2_-activated PKCδ is responsible for its pro-apoptotic actions. Transfection of the catalytic fragment of PKCδ (PKCδ^CAT^) induced apoptosis in BG1 cells. PKCδ^CAT^ alone could induce cleavage of caspase-3 and also increase phH2Bser14 indicating that catalytic cleavage of PKCδ, leading to the liberation of PKCδ^CAT^ fragment is necessary for the pro-apoptotic effects.

p38 MAPK regulates a number of cellular processes in response to a variety of stress signals [67] such as UV radiation and inflammatory cytokines. In our previous study, we reported that 2MeOE_2_ treatment induces p38 phosphorylation and a selective p38 inhibitor, SB203580 [68], reduces its pro-apoptotic effect in the human ovarian cancer cells [14]. Evidence suggests that catalytically cleaved PKCδ can phosphorylate p38 MAPK in smooth muscle cells [69, 70], fibroblasts [71], prostate cancer cells [72] and hepatic stellate cells [73]. In our current study, we have also investigated whether 2MeOE_2_ mediated phosphorylation of p38 MAPK involves PKCδ signaling. Following 2MeOE_2_ treatment, PKCδ^WT^ transfection shows a significantly higher amount of phosphorylated p38 MAPK. 2MeOE_2_ treatment of PKCδ-depleted BG1 cells had decreased amount of phosphorylated p38 MAPK compared to scrambled siRNA control. These data suggest that 2MeOE_2_ mediated activation of PKCδ also phosphorylates p38 MAPK and the functional C3 domain of PKCδ is required for this activity. PKCδ^CAT^ alone successfully increased phosphorylation of p38 MAPK in BG1 cells. SB203580 reduced the pro-apoptotic effects of PKCδ^CAT^. Previously, we reported that SB203580 reduces the amount of caspase-3 cleavage induced by 2MeOE_2_ in human ovarian cancer cells [14]. In contrast, SB203580 almost entirely inhibited downstream caspase-3 activation by PKCδ, indicating that p38 MAPK pathway is a principal pathway through which PKCδ-mediated apoptotic effects are elicited. Interestingly, SB203580 significantly reduced the phH2Bser14 expression induced by PKCδ^CAT^, although it was still significantly higher than pGFP transfected or SB203580 treated pGFP cells. This suggests that p38 MAPK and subsequent activation of caspase-3 plays a major role in cleavage and activation of more PKCδ. The catalytic activation of PKCδ therefore results in a feedforward cycle amplifying the apoptotic signal.

Transfection of PKCδ^DN^ significantly reduced cleavage of caspase-3 and γH2Ax expression compared to their respective pGFP controls, suggesting that the pro-apoptotic actions of 2MeOE_2_ are partially dependent on PKCδ activation. The phH3ser10 expression was lower in 2 μg PKCδ^DN^ transfected cells however there was no significant alteration in the groups receiving higher amounts of PKCδ^DN^. Therefore no discernible pattern was observed with phH3ser10. Interestingly, when we co-transfected PKCδ^DN^ in an increasing amount (2 μg, 3 μg and 4 μg) with the same amount of PKCδ^CAT^ (2 μg), we found that PKCδ^DN^ transfected cells had lower cleavage of caspase-3 and phH3ser10 expression compared to their respective pGFP controls. Notably, γH2Ax expression was found unaltered in these groups suggesting that γH2Ax is not directly affected by PKCδ. This also corroborates the role of PKCδ in the apoptotic phH3ser10 expression.

**Figure 7:**
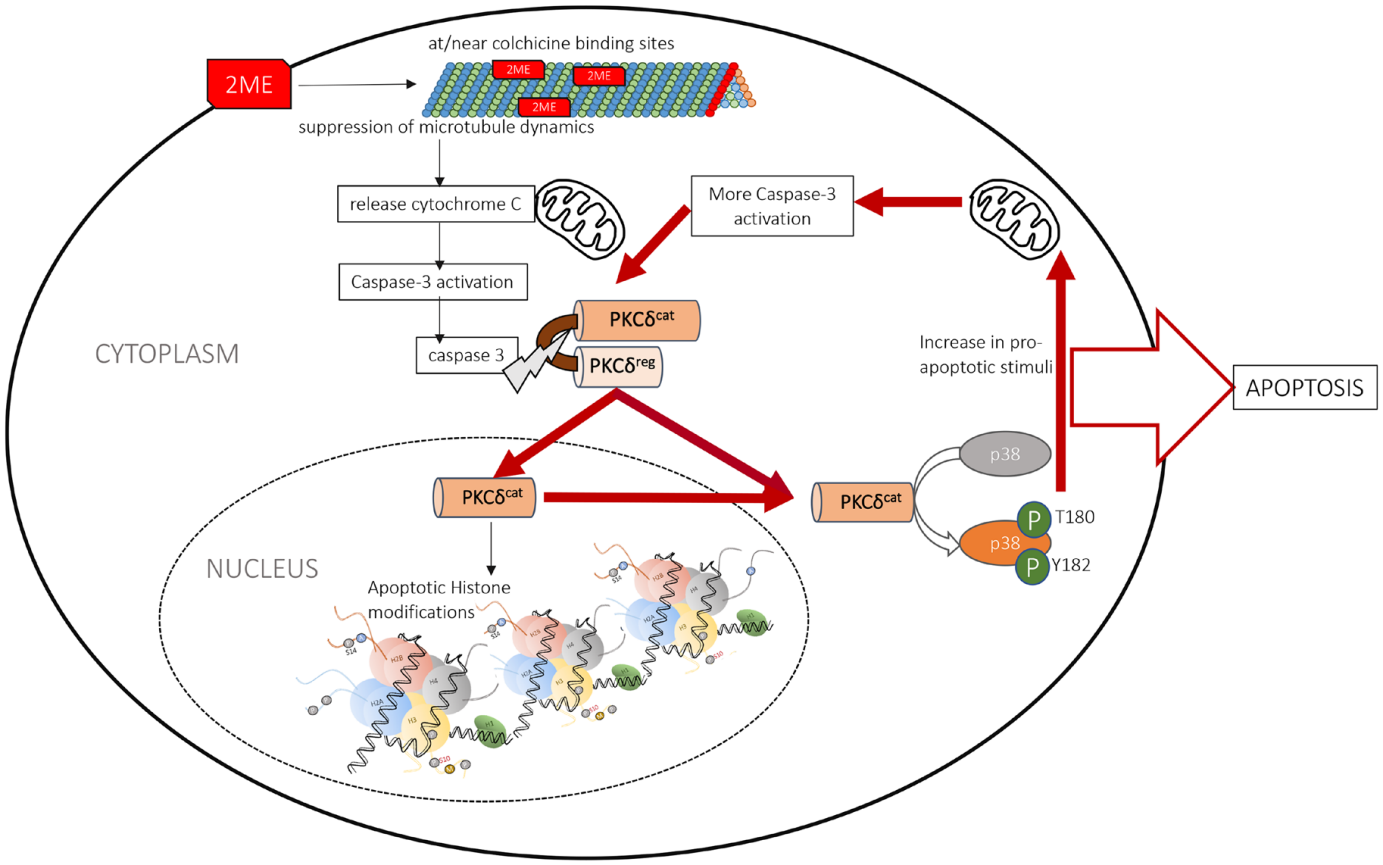
Schematic diagram of the molecular action of 2MeOE_2_. 2MeOE_2_ -mediated pro-apoptotic actions involve the catalytic activation of PKCδ by cellular caspases. The catalytic fragment of PKCδ is responsible for the apoptotic histone modifications in the nucleus and activates p38 MAPK pathway in the cytosol, which induces more activation of caspases that consequently cleave and activate more PKCδ. Therefore this cycle accelerates and amplifies the 2MeOE_2_-mediated apoptotic signal.

Our findings indicate that 2MeOE_2_ -mediated anti-tumor actions involve the catalytic activation of PKCδ in the pro-apoptotic pathway. The catalytic fragment of PKCδ is responsible for the apoptotic histone modifications and acceleration of the apoptotic cascade through p38 MAPK pathway. Dietary flaxseed supplementation activates PKCδ secondary to increasing endogenous production of 2MeOE_2_ which drives apoptosis in ovarian cancer cells (Figure 7). This study offers new insight into the molecular underpinnings of dietary flaxseed’s chemopreventative actions in ovarian cancer.

## MATERIALS AND METHODS

### Materials

BG1 cells were obtained from the laboratory of Dr. Ken Korach at NIEHS and Hey C2 cell line was obtained from Dr. Jean Hurteau at Northshore University Health, Evanston Hospital. TOV112D (CRL11731) cell line was purchased from ATCC. HyClone DMEM culture media (with and without phenol red) from ThermoFisher (SH30604.02); 2-methoxyestradiol from Sigma-Aldrich (M6383); 100× HALT protease and phosphatase inhibitor cocktail from ThermoFisher (78440); DyLight™800 conjugated goat anti-rabbit IgG antibody (H&L) (35571) and DyLight™680 conjugated goat anti-rabbit IgG antibody (H&L) (35518) from Thermofisher. Alexa-594 donkey anti-rabbit secondary (133200) from Jackson Immuno Research. jet-PEI^®^ purchased from Polyplus transfection (catalog no.101-10). Expression plasmids encoding PKCδ^WT^ (Addgene plasmid # 16386), PKCδ^DN^ (Addgene plasmid # 16389), and PKCδ^CAT^ (Addgene plasmid # 16388) were gifts from Bernard Weinstein [74], pSUPER-PKCδ^RNAi^ (Addgene plasmid # 10819) was a gift from Alex Toker [75] and pSUPER-scramble (Addgene plasmid # 118349) was a gift from Lea Sistonen [76]. E. Z. N. A.^®^ plasmid maxi kit (D6922-02) was purchased from Omega Bio-Tek. SB203580 p38 MAPK inhibitor was purchased from Cayman Chemical (13067).

### Cell culture and treatments

BG1, HeyC2 and TOV112D cells were cultured in DMEM (with phenol red) media supplemented with 10% fetal bovine serum and 7500 IU penicillin, 7500 μg streptomycin, incubated at 5% CO_2_ and 37°C. Cells were seeded with a density of 4 × 10^5^ cells per well in 6-well tissue culture plates. Media was changed after 24 hours to DMEM (phenol red-free) supplemented with 10% charcoal stripped newborn calf serum and 0.75% of 10,000 μg/ml penicillin-streptomycin. 2MeOE_2_ (stock in DMSO) dilutions were prepared in phenol red-free DMEM before adding to the cells. Following a 24 h incubation, cells were photographed harvested and total proteins were extracted.

### Protein isolation from cells

Cells were scraped off after adding 200 μl ice-cold PBS and collected in Eppendorf tubes. Tubes were centrifuged at 2000 g at 4°C for 3 min, supernatant discarded and the pellets were resuspended in 30 μl of protein lysis buffer (1× HALT protease and phosphatase inhibitor cocktail in 0.1% SDS/1xPBS). Following a short burst of sonication, protein quantities were estimated by BCA method and stored at −20°C.

### Immunocytochemistry

Cells were cultured on cover glass (2 × 10^4^ cells per cover glass) and treated after 24 h of initial seeding. Following treatment, cells were fixed with 1:1 methanol: acetone (v/v) for 20 mins. Samples were then blocked with 5% bovine serum albumin for 30 mins and incubated with primary antibodies (1:100 dilution in blocking solution) overnight at 4°C (Table 1). Following 1x PBS rinse, secondary antibodies (Alexa Fluor^®^ 594 nm, red, 1:200 and Alexa Fluor^®^ 488 nm, green, 1:200, diluted in blocking solution) were applied for a 1 h incubation at room temperature. Samples were then washed with 1× PBS and mounted with DAPI Fluoromount G (Southern Biotech). Slides were then visualized with a Leica DM5500Q microscope, images were captured with a Leica DFC365FX camera, taken from A4 (DAPI), Alexa 488 nm (green) and Alexa 594 nm (red) channels. Images from multiple channels were superimposed using the Leica Application Suite-Advanced Fluorescence version 2.6.0.7266 software.

**Table 1:** List of primary antibodies

Target protein	Manufacturer	Raised in	Dilutions
Caspase-3	Cell signaling technology, 9665S	Rabbit	1:500
Cleaved caspase-3	Cell signaling technology, 9664S	Rabbit	1:500
p38 MAPK	Cell signaling technology, 9212S	Rabbit	1:700
Phospho p38 MAPK	Cell signaling technology, 4511S	Rabbit	1:700
Phospho histone H3 (ser10)	Cell signaling technology, 9701S	Rabbit	1:1000
Phospho histone H2B (ser14)	Cell signaling technology, 6959S	Rabbit	1:1000
*γ*H2Ax	Novus Bio, NBP1-19255	Mouse	1:1000
Protein kinase Cδ	Cell signaling technology, 2058S	Rabbit	1:1000
β-actin	Cell signaling technology, 3700S	Mouse	1:1000

### Bacterial culture and plasmid DNA preparation

DH5α cells transfected with respective plasmids were grown in standard LB broth at 37°C with 100 μg/ml ampicillin. Plasmid DNA was prepared from 200 ml. overnight grown bacterial culture with E.Z.N.A. plasmid maxi kit following manufacturer’s protocol. Extracted DNA was purified by re-precipitation with 0.1 volume of 3M Na-acetate (pH 5.2) and 0.7 volume of isopropanol at −20°C overnight. Final DNA pellet was resuspended in 1× TE buffer, concentration determined by Nanodrop 2000 (Thermo Scientific).

### Transient transfection

BG1 cells were seeded in 6-well tissue culture plates (2 × 10^4^ cells per well). Medium was replaced on the next day with DMEM (with phenol red) without antibiotics and transfection was performed with jet-PEI. Per well, 2 μg of plasmid DNA was added to 200 μl of Opti-MEM media; then 6 μl of jet-PEI (1 μg/μl stock) was added; the mastermix was vortexed every 5 mins for 20 mins and added to respective wells. Transfected cells were cultured for 24 h at 5% CO_2_ and 37°C. At this point, cells were either harvested or treated for further analysis.

### Western blot analysis

Western blot was performed as described previously. 30 μg of total protein was resolved using an SDS-PAGE gel and transferred to a PVDF membrane. Membranes were blocked by Sea Block blocking buffer (Pierce) for an hour at RT, followed by overnight incubation at 4°C with the primary antibodies diluted in the blocking buffer (Table 1). Membranes were washed with 1×TBS with 0.01% Tween-20 followed by an hour incubation at room temperature with an anti-mouse Dylight 680 and anti-rabbit Dylight 800 secondary antibodies (1:2000 dilution in 1×TBST with 0.01% Tween-20). After washing the membranes with 1x TBST with 0.01% Tween-20, the membranes were imaged in Odyssey CLx imaging system (Li-COR Biosciences). All target proteins were normalized to β-actin expression.

### Statistical analysis

Statistical analysis was performed by GraphPad Prism v5.0. Unpaired student’s *t*-test were performed for analysis of two groups. One-way or Two-way analysis of variance (ANOVA) was performed for multiple groups followed by Tukey’s range test. Statistically significant change was considered for a *p* value of < 0.05.

## SUPPLEMENTARY MATERIALS



## Abbreviations

EOC: Epithelial ovarian cancer
2MeOE_2_: 2-methoxyestradiol
PKCδ: Protein kinase C delta
CYP: Cytochrome P450
MAPK: Mitogen-activated protein kinase
GFP: Green fluorescent protein
DMEM: Dulbecco’s modified Eagle’s medium
DMSO: Dimethyl sulfoxide
PBS: Phosphate buffered saline
DAPI: 4′,6-diamidino phenylindole
PEI: Polyethylenimine
SDS-PAGE: Sodium dodecyl sulphate polyacrylamide gel electrophoresis
PVDF: Polyvinylidene difluoride
