# Supplementary material for: The pro-apoptotic actions of 2-methoxyestradiol against ovarian cancer involve catalytic activation of PKCδ signaling
Source: Oncotarget. 2020 Oct 6;11(40):3646–59. doi: 10.18632/oncotarget.27760 (PMC7546757; doi:10.18632/oncotarget.27760)
Supplement: Supplementary file 1 [file oncotarget-11-3646-s001.pdf]

The pro-apoptotic actions of 2-methoxyestradiol against ovarian cancer involve catalytic activation of PKCδ signaling

SUPPLEMENTARY MATERIALS

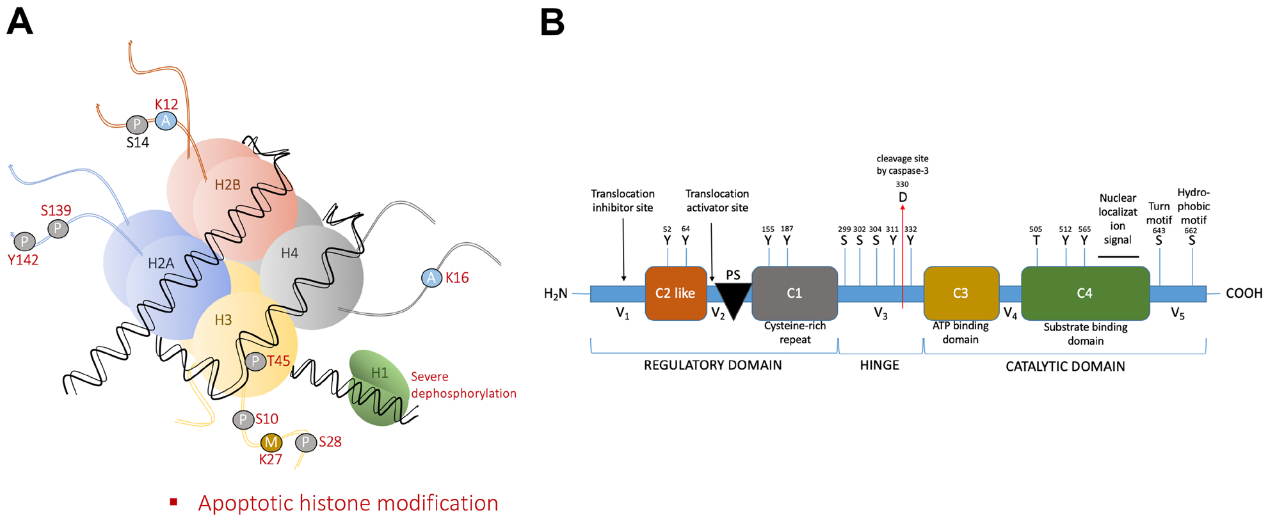

Supplementary Figure 1: (**A**) Schematic diagram describing apoptotic histone modifications (red). (**B)** Diagram depicting different domains and phosphorylation sites of the full length human PKCδ.
